# Effect of core training on skill-related physical fitness performance among soccer players: A systematic review

**DOI:** 10.3389/fpubh.2022.1046456

**Published:** 2023-01-05

**Authors:** Shengyao Luo, Kim Geok Soh, Lingling Zhang, Xiuwen Zhai, Jaka Sunardi, Yongqi Gao, He Sun

**Affiliations:** ^1^Department of Sports Studies, Faculty of Educational Studies, Universiti Putra Malaysia, Seri Kembangan, Malaysia; ^2^Department of Football Teaching, Faculty of Football College, Shandong Sport University, Jinan, Shandong, China; ^3^Department of Language and Humanities Education, Faculty of Educational Studies, Universiti Putra Malaysia, Seri Kembangan, Malaysia; ^4^Faculty of Sport Science, Universitas Negeri Yogyakarta, Yogyakarta, Indonesia

**Keywords:** core training, soccer players, physical fitness, skill, effect

## Abstract

**Aims:**

This study aims to present an in-depth review of the available literature on the effect of core training on skill-related physical fitness performance among soccer players, as well as to offer suggestions for researchers and coaches.

**Methods:**

The data in this study were presented based on the Preferred Reporting Items for Systematic Reviews and Meta-Analyses (PRISMA) guideline. Using scientific databases and web search engines including Scopus, Ebscohost, Web of Science, PubMed, and Google Scholar, researchers collected studies from the published literature. Only 26 of the 84 articles satisfied all the inclusion criteria and were thus included in the systematic review. The quality of each study was determined using the PEDro scale. The scores for 26 studies range between three and six.

**Results:**

Core training can improve soccer players' skill-related physical fitness, including their power, speed, balance, and agility.

**Conclusion:**

The core is the anatomic and functional center of the body as well as its “engine.” All movements emanate from the center of the body and are transmitted to the extremities. The core muscles differ from the limb muscles because they frequently cocontract, thus making the torso hard to the point whereby all the muscles work together to become synergists. Theoretically, a strong core permits the passage of force from the lower body to the upper body with minimal energy loss in the torso. Based on the 26 studies, this review suggests that core training should be incorporated into the daily training sessions of soccer players, with a minimum frequency and length of 15 min per training session, twice per week, for 4 weeks.

**Systematic review registration:**

https://inplasy.com, identifier INPLASY202290045.

## 1. Introduction

Soccer, which is among the world's most popular sports (1), is played by a large number of men and women with varying skill levels. Stølen et al. (2) asserted that technical, tactical, physical, physiological, and mental elements primarily affect soccer players' overall performance. Moreover, many researchers have shown that, among the subfactors mentioned above, physical fitness has the greatest influence on soccer players' overall performance (2–4). In every sporting activity, an exceptional level of physical fitness is required for the efficient understanding, enhancement, and execution of athletic skills (5–7).

Indeed, soccer players must remain in peak physical condition. Physical fitness has been described in several ways. The American Academy of Physical Education adopted the following definition of physical fitness: the capacity to perform daily tasks with energy and alertness, without undue fatigue, and with sufficient energy to engage in leisure activities and withstand the above-average physical stress encountered in emergencies (8). Researchers opined that physical fitness might be divided into two categories: health-related physical fitness and motor skill-related physical fitness, based on their ongoing research on the subject. Health-related physical fitness refers to fitness characteristics that have a strong correlation with beneficial health outcomes such as body composition, cardiovascular endurance, flexibility, muscular strength, and endurance (9, 10). In contrast, motor skill-related physical fitness comprises physical fitness and motor skills that enhance athletic performance, such as agility, balance, coordination, speed, power, and reaction time (11). A few studies have shown that a high level of physical fitness enables football players achieve the highest possible performance (12–14). Sarmento et al. (15) identified that elite soccer players typically run between nine and 14 km during a 90-min game, with 22–24% of the total match distance covered at speeds higher than 15 km/h (high-intensity threshold); 8–9% at speeds higher than 20 km/h (very high-intensity threshold); and 2–3% at speeds higher than 25 km/h (sprinting threshold). A study indicated a 2% increase in the total distance covered and a 30% increase in high-intensity sprints in the modern game of soccer. Consequently, soccer matches are becoming more physically taxing (16).

D'Isanto et al. (17) concluded that strength training is the most significant component of an athlete's performance. Another researcher suggested that core training—a new strength-training method—improves strength transmission, coordinated combination, and muscle control ability (18). It embodies the new concept of whole-body integrity and multi-muscle groups simultaneously participating in sports in multiple dimensions (18). In fact, core training, which integrates multiple muscle groups, requires greater coordination, which may improve strength-power adaptation and, consequently, on-field performance (18, 19). Core muscles can be considered synergists for these muscles, and play a significant role in efficient biomechanics (20, 21). During integrated athletic activities, these muscles can produce excellent force, transference, and control of the terminal section. Defined as the pre-programmed integration of local, single-joint, and multi-joint muscles, core muscle activities can provide stability and produce motion (20). This results in proximal stability for distal mobility, proximal to distal patterning of force generation, and the formation of interactive moments that move and protect the distal joints. Additionally, interactive moments maximize force at the distal end, as well as maintain precision and stability at the distal tip (20). This may explain why football players might benefit from core training.

Anatomically, the core comprises the lumbar spine, abdominal wall muscles, back extensors, and quadratus lumborum. The multi-joint muscles latissimus dorsi and psoas, which travel through the core and connect it to the pelvis, legs, shoulders, and arms, are also included (21). More specifically, the thoracolumbar fascia is not only a crucial structure that connects the lower limbs (*via* the gluteus maximus) to the upper limbs (*via* the latissimus dorsi) (20), it is also attached to the internal oblique muscle and transversus abdominis, which results in three-dimensional support to the lumbar spine and facilitates core stability (22). It enables forming a ring around the abdomen to create a stable corset effect—this ring comprises the posterior fascia, anterior fascia, and ventral muscles (23). Moreover, core muscles comprise two types of muscle fibers: slow-twitch and fast-twitch fibers. The former mainly comprises the local muscle system (deep muscle layer) (24). These muscles are shorter and more suitable for controlling intersegmental motion and responding to changes in posture and extrinsic loads. Key local muscles include the transversus abdominis, multifidi, internal oblique, deep transversospinalis, and pelvic floor muscles. In contrast, fast-twitch fibers comprise the global muscle system (superficial muscle layer). These muscles are longer and have larger lever arms, which enables substantial production of torque and movement. Key global muscles include the erector spinae, external oblique, rectus abdominis, and quadratus lumborum (24). Numerous studies have supported the notion that the core is the anatomical and functional center of an organism and its engine. All motions originate at the center and are transmitted to the extremities (25, 26). However, it cannot be ignored that the gluteal muscles are also key power generators (27). The core musculature differs from the limb musculature in that the core muscles frequently co-contract, hardening the torso to the point where all muscles become synergists (21). It has been hypothesized that a strong core facilitates the passage of force from the lower to the upper body, with minimum energy dissipation in the torso (27, 28). Running, jumping, and throwing are adversely impacted if power is generated but not transferred (29). Despite some evidence supporting the fact that athletes benefit from core training, no study has yet summarized the information on the effect of core training on skill-related physical fitness among soccer players. Therefore, this systematic review aims to investigate the effects of core training on skill-related physical fitness performance among soccer players.

## 2. Methods

### 2.1. Protocol and registration

This review followed the Preferred Reporting Items for Systematic Reviews and Meta-Analyses (PRISMA) guidelines for data collection as well as for the selection and analysis of existing studies. It is registered on the INPLASY website (https://inplasy.com—Registration) Number: INPLASY202290045; 10.37766/inplasy2022.9.0045—as recommended by Moher et al. (30).

### 2.2. Search strategy

Several scientific journal indices were used to search the existing literature, such as Scopus, Ebscohost, Web of Science, and PubMed. The search was also conducted on Google Scholar and the bibliography section of relevant studies until August 2022. For each index, a search was performed using the titles and abstracts of articles. The main keywords for gathering the relevant articles were as follows: (“Core Strength Training” OR “Core-Muscle Training” OR “Core training” OR “Core-Stability Exercise” OR “Core Exercise”) AND (“Physical Fitness” OR “Physical Performance” OR “Skill-Related Physical Fitness” OR “Agility” OR “Balance” OR “Coordination” OR “Power” OR “Speed” OR “Reaction Time”) AND (“Soccer Players” OR “Soccer Athletes” OR “Football Players” OR “Football Athletes”).

### 2.3. Eligibility criteria

A literature search was performed using the PICOS framework (Table 1). The selected articles had to be in English and from academic journals. Meanwhile, PICOS refers to (1) population, (2) intervention, (3) comparison, (4) outcome, and (5) research design. Each PICOS component was used as an inclusion criterion for the retrieved studies. The following criteria must be satisfied for each study:

i. The study population comprised healthy football players without any sports injuries, irrespective of sex or age.
ii. The intervention of core training was involved.
iii. The comparison in the study included a core training group vs. non-core training group, comparisons between different types of core training groups, and within-group comparisons with a single core training group.
iv. The study results included at least one skill-related physical fitness performance.
v. Studies involved empirical experiments, including non-randomized controlled trials, randomized controlled trials, and non-randomized non-controlled trials.

**Table 1:** Criteria from PICOS framework.

**PICOS**	**Detailed information**
Population	Healthy football players
Intervention	Core training
Comparison	Core training group vs. non-core training group; Comparisons between different types of core training groups; Within group comparisons with a single core training group.
Outcome	Include core training with various kinds of skill-related physical fitness performance among players
Study design	Non-randomized controlled trials, randomized controlled trials, and non-randomized non-controlled trials

### 2.4. Study selection

Two authors selected and retrieved the articles that met the inclusion criteria. The EndNote citation management system was used to remove duplicates. The titles and abstracts were evaluated to identify articles that could be included in this study. In the event of disagreement between the two authors (Luo and Zhang) over the selection of an article, a third author (Soh) was consulted to review the entire work and make the ultimate determination.

### 2.5. Data extraction and quality assessment

The following data were extracted from qualified articles post-screening: (1) author name and publication year; (2) population characteristics such as number, sex, level, and age; (3) intervention characteristics such as measures index, type, frequency, and duration; and (4) the final research results.

The PEDro scale is a reliable and precise tool for measuring the quality of the method used to produce a systematic review (31). This scale comprises 11 items, with points ranging from 0 to 10. Two independent raters evaluated these 11 items using “yes” (1 point) or “no” (0 point). Scoring discrepancies were resolved with the help of a third rater. However, as it is related to external efficacy, the eligibility criteria score was factored into the final score. The quality of the procedures was greater with a higher score. The authors state that an overall PEDro scores of 0–3 is deemed “poor,” 4–5 is deemed “fair,” 6–8 is deemed “good,” and 9–10 is deemed “excellent.” However, these categories require further investigation. Moreover, the optimal total PEDro score for trials evaluating complex interventions (e.g., exercise) is 8–10 (32).

## 3. Results

### 3.1. Study selection

**Figure 1:**
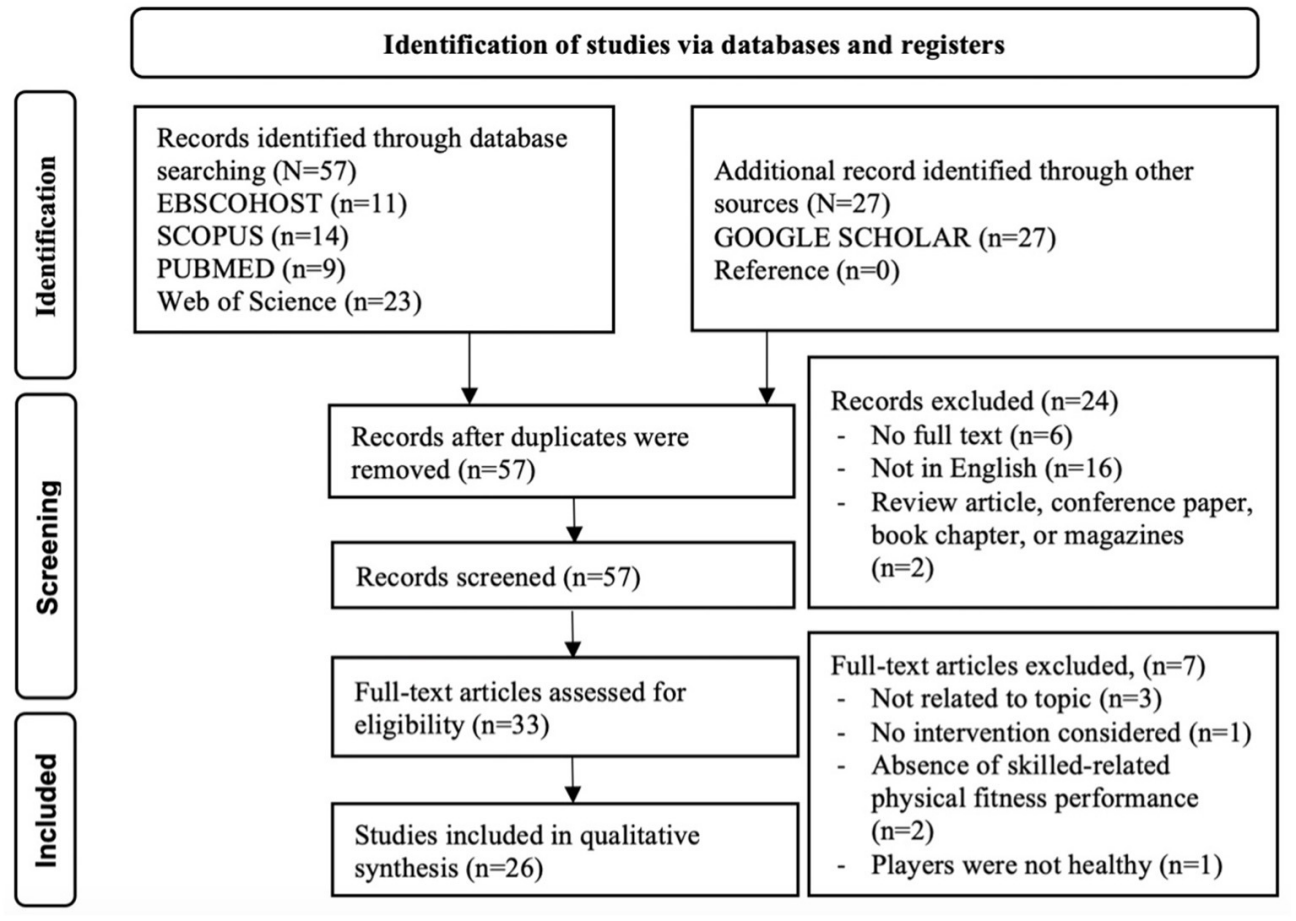
The search, screening, and selection processes for suitable studies - based on PRISMA guideline.

Figure 1 shows the techniques used to evaluate the literature. Initially, this study identified 57 articles from different databases, including EBSCOHOST (*n* = 11), SCOPUS (*n* = 14), PUBMED (*n* = 9), and Web of Science (*n* = 23), and 27 articles from other sources, including GOOGLE SCHOLAR (*n* = 27) and reference (*n* = 0). Following the screening process, 57 potentially eligible articles were identified. This was performed after the Endnote software deleted duplicate articles. The second round of filtering comprised six articles lacking full text, 16 articles not written in English, and two articles not from journals. The eligibility of 33 full-text publications was reviewed during the third screening step. Seven publications were omitted because three were irrelevant to the issue, two did not investigate skill-related physical fitness performance, one lacked an intervention, and one included unhealthy athletes. In total, 26 relevant studies satisfied the inclusion criteria and were considered for qualitative synthesis.

### 3.2. Study quality assessment

The scores for each study are listed in Table 2 based on their PEDro scale scores. All the studies scored between three and six on the PEDro scale. Owing to the requirements of secret allocation and blinding participants, assessors, and therapists, all trials were penalized in terms of points. Participants, evaluators, and therapists are difficult to blind because the intervention includes strength training, which is accompanied by professionalism and risk of sports injury.

**Table 2:** Summary of methodological quality evaluation scores.

**Study**	**Eligibility criteria**	**Random allocation**	**Allocation concealment**	**Baseline comparability**	**Blind participants**	**Blind therapist**	**Blind assessor**	**Follow-up**	**Intention to treat analysis**	**Between group comparisons**	**Point measure and variability**	**Total PEDro score**
Dogan et al. (33)	1	0	0	1	0	0	0	1	1	1	1	5
Yoka et al. (34)	0	0	0	0	0	0	0	1	1	1	1	4
Gasim et al. (35)	1	1	0	0	0	0	0	1	1	1	1	5
He (36)	0	0	0	0	0	0	0	1	1	1	1	4
Lago-Fuentes et al. (37)	1	1	0	1	0	0	0	1	1	1	1	6
Mossa (38)	1	1	0	1	0	0	0	1	1	1	1	6
Genç and Cigerci (39)	0	0	0	1	0	0	0	1	1	1	1	5
Koçak et al. (40)	0	0	0	0	0	0	0	1	1	0	1	3
Taskin (41)	0	0	0	1	0	0	0	1	1	1	1	5
Yapici (42)	1	0	0	1	0	0	0	1	1	1	1	5
Bavli and Koç (43)	1	1	0	1	0	0	0	1	1	1	1	6
Afyon (44)	1	0	0	1	0	0	0	1	1	1	1	5
Turna (45)	1	0	0	1	0	0	0	1	1	1	1	5
Atli (46)	1	0	0	1	0	0	0	1	1	1	1	5
Sever and Zorba (47)	1	1	0	1	0	0	0	1	1	1	1	6
Brull-Muria and Beltran-Garrido (48)	1	0	0	1	0	0	0	1	1	1	1	5
Alemayehu et al. (49)	0	1	0	1	0	0	0	1	1	1	1	6
Mendes (50)	0	0	0	0	0	0	0	1	1	0	1	3
Bayrakdar et al. (51)	1	0	0	1	0	0	0	1	1	1	1	5
Boyaci and Afyon (52)	0	0	0	1	0	0	0	1	1	1	1	5
Afyon et al. (53)	0	0	0	1	0	0	0	1	1	1	1	5
Wang (54)	0	0	0	1	0	0	0	1	1	1	1	5
Zhou et al. (55)	0	0	0	0	0	0	0	1	1	1	1	4
Iacono et al. (56)	1	0	0	1	0	0	0	1	1	1	1	5
Prieske et al. (57)	0	1	0	1	0	0	0	1	1	1	1	6
Doganay et al. (58)	1	1	0	1	0	0	0	1	1	1	1	6

### 3.3. Participant characteristics

Table 3 shows the characteristics of the 26 articles that satisfied all inclusion criteria. The properties are as follows: (1) Athletes. Among the 26 articles, four investigated athletes at the university level (33–35, 55); four articles investigated the elite level (accomplished athletes in non-professional contexts) (36, 48, 56, 57); one investigated athletes at the professional level (37); six investigated athletes at the amateur level (41–44, 46, 53); one investigated athletes at the semi-professional level (59); one investigated athletes at the junior level (49); one investigated athletes at the higher school level (50); and one article investigated athletes at the secondary school level (51). However, seven articles did not report the number of participants (38–40, 45, 52, 54, 58). (2) Number, sex, and age. The total number of participants was 912: 344 males, 74 females, and the remaining 494 participants were mixed, with no sex-based statistics. Except for two articles, all others recorded participants' age (36, 54). The oldest and youngest participants were 30 and 9 years of age, respectively; the average age was 17.6 years.

**Table 3:** Population, intervention, and main outcome.

**Study**	**Population**				**Intervention**			**Main outcome related to physical fitness**
	***N***	**Level of athletes**	**Sex**	**Age**	**Type**	**Physical fitness measured index**	**Frequency and duration**	
Dogan et al. (33)	44	University	Male	18–30	EG: core training CG: normal training	Vertical jump, 20 m speed	90 min 2 times/week 8 weeks	Vertical jump ↑ 20 m speed ↑
Yoka et al. (34)	42	University	Male	18–25	G 1: core training (footballer) G 2: football training G 3: core training (sedentary) G 4: no training (sedentary)	The biodex balance system static balance, dynamic balance	60 min 2 times/week 10 weeks	The biodex balance system static balance ↑ Dynamic balance ↑
Gasim et al. (35)	18	University	Male	17–18	Group 1: core training Group 2: plyometric training Group 3: soccer group	Y-balance test, dynamic balane	2 times/week 8 weeks	balance test ↑ Dynamic balance ↑
He (36)	44	Elite	Mixed (22 F; 22 M)	No report	EG 1: core training (male) EG 2: core training (female) CG 1: football training (male) CG 2: football training (female)	Balance	No report	Balance ↑
Lago-Fuentes et al. (37)	14	Professional	Female	18–28	EG: core strength training (stable) CG: core strength training (unstable)	10 m sprint, repeated sprint ability test. vertical jump	3 times/week 6 weeks	10 m sprint ↑ Repeated sprint ability test ↑ Vertical Jump ↔
Mossa (38)	26	No report	No report	14	EG: core strength exercise CG: regular soccer training	Vertical jump, the illinois agility test, *t*-test, 10 m dash, 40 m dash	2 times/week 12 weeks	Vertical jump ↑ The illinois agility test ↑ *T*-test ↑ 10 m dash ↑ 40 m dash ↑
Genç and Cigerci (39)	20	No report	No report	12-14	EG: core training CG: routine soccer training	10 m speed, 30 m speed, standing long jump	60 min 3 times/week 8 weeks	10 m speed ↔ 30 m speed ↔ Standing long jump ↑
Koçak et al. (40)	15	No report	Male	18–21	EG: core training	35 m repeated sprint (6 times)	40 min 3 times/week 8 weeks	35 m repeated sprint (6 times) ↑
Taskin (41)	40	Amateur	Female	18–19	EG: core training CG: routine soccer training	10 m speed test, 30 m speed test, vertical jump, standing long jump	3 times/week 8 weeks	10 m speed test ↑ 30 m speed test ↑ Vertical jump ↑ Standing long jump ↑
Yapici (42)	32	Amateur	Male	13–14	EG: core exercise CG: normal training	Standing long jump, zigzag agility test, 10 m sprint, 20 m sprint	6 weeks	Standing long jump ↑ Zigzag agility test ↑ 10 m sprint ↑ 20 m sprint ↑
Bavli and Koç (43)	18	Amateur	Male	15 ± 0.8	EG 1: dynamic core exercise EG 2: static core exercise CG: soccer training	Standing long jump	75 min 8 weeks	Standing long jump ↑
Afyon (44)	36	Amateur	No report	19–23	EG: core training CG: football training	Vertical jump	2 times/week 8 weeks	Vertical jump ↑
Turna (45)	30	No report	Male	9–11	EG: core training CG: conventional training	Vertical jump, 30 m sprint, flamingo balance	2 times/week 6 weeks	Vertical jump ↑ 30 m sprint ↑ Flamingo balance ↑
Atli (46)	40	Amateur	No report	18–24	EG: core training CG: football training	Vertical jump, 30 m sprint, Illinois agility test	15 min 3 times/week 6 weeks	Vertical jump ↑ 30 m sprint ↑ Illinois agility test ↑
Sever and Zorba (47)	38	Semi- 48 professional	No report	16–20	EG 1: dynamic core exercise EG 2: static core exercise CG: football training	505 agility, arrowhead agility, 10 m dash, 30 m dash, standing long jump, vertical jump	Over 8 h/week 3 times/week 8 weeks	505 agility ↔ Arrowhead agility ↔ 10 m dash ↔ 30 m dash ↔ Standing long jump ↔ Vertical jump ↔
Brull-Muria and Beltran-Garrido (48)	14	Elite	Male	16–18	EG: core training CG: usual training	10-m sprint, V-cut	20 min 2 times/week 8 weeks	10-m sprint ↑ V-cut ↑
Alemayehu et al. (49)	26	Junior	Male	Under 17	EG: core training CG: regular soccer training	Vertical jump, standing long jump, *t*-test, Illinois agility test, 10 m dash, 40 m dash	35–45 min 2 times/week 12 weeks	Vertical jump ↑ Standing long jump ↑ *T*-test ↑ Illinois agility test ↑ 10 m dash ↑ 40 m dash ↑
Mendes (50)	44	Higher school	No report	18–30	EG: core training	10 m sprint, 20 m sprint, zigzag test	60 min 2 times/week 6 weeks	10 m sprint ↑ 20 m sprint ↑ Zigzag test ↔
Bayrakdar et al. (51)	30	Secondary school	No report	12–14	EG 1: dynamic core exercise EG 2: static core exercise CG: football training	30 m speed, standing long jump, vertical jump, arrowhead agility, 505 agility	30 min 2 times/week 9 weeks	30 m speed ↑ Standing long jump ↑ Vertical jump ↑ Arrowhead agility ↑ 505 agility ↑
Boyaci and Afyon (52)	40	No report	No report	12–14	EG: core training CG: normal training	Vertical jump, standing long jump, flamingo balance test, 20 m sprint, throwing medicine ball test while sitting	25–30 min 2 times/week 12 weeks	Vertical jump ↑ Standing long jump ↑ Flamingo balance test ↑ 20 m sprint ↑ Throwing medicine ball test while sitting ↑
Afyon et al. (53)	40	Amateur	No report	22–23	EG: core training CG: regular training	30 m sprint, *t*-drill agility test, Illinois agility test	30 min 2 times/week 8 weeks	30 m sprint ↑ *T*-drill agility test ↑ Illinois agility test ↑
Wang (54)	160	No report	No report	No report	EG: core training CG: traditional training	100, 30 m sprint, standing long jump	4 h/week 32 weeks	100 m ↑ 30 m sprint ↑ Standing long jump ↑
Zhou et al. (55)	18	University	No report	19–21	EG: core strength training CG: normal training	20 m starting run, vertical jump, Illinois agility test	4 times/week 32 weeks	20 m starting run ↑ Vertical jump ↑ Illinois agility test ↑
Iacono et al. (56)	20	Elite	Male	Under 19	EG: core strength training CG: normal training	Static balance, dynamic SEBT balance test	5 times/week 4 weeks	Static balance ↑ Dynamic SEBT balance test ↑
Prieske et al. (57)	39	Elite	Male	17 ± 1	EG: core strength training (stable) CG: core strength training (unstable)	Vertical jump, 20-m sprint, *t* agility test	2–3 times/weeks 9 weeks	Vertical jump ↔ 20-m sprint ↑ *T* agility test ↔
Doganay et al. (58)	24	No report	Male	17–18	EG: core training CG: usual training	40 m speed, hexagon test, *t* agility test	30–35 min 3 times/week 8 weeks	40 m speed ↑ Hexagon test ↔ *T* agility test ↑

EG, experimental group; CG, control group; G, group; SEBT, star excursion balance test; ↑, significant within-group improvement from pre-test to post-test; ↔, non-significant within-group change from pre-test to post-test.

### 3.4. Intervention characteristics

Table 3 provided detailed information on 26 studies related to intervention characteristics, mainly including type, frequency, and duration. As for type, most studies used core training as an intervention; however, a few referred to core training as core strength training (37, 55–57), core strength exercise (38), core exercise (42), or dynamic or static core exercise (43, 51, 59).

The frequency in all studies ranged from twice a week to five times a week. Specifically, interventions in 12 studies were performed twice a week (33–35, 38, 44, 45, 48–53), seven studies applied interventions thrice a week (37, 39–41, 46, 58, 59), one applied intervention four times a week (55), one applied intervention five times a week (56), and one study applied intervention twice or thrice a week (57). Meanwhile, the exercise duration for each practice in most studies ranged from 15 to 90 min. The training duration in studies was 90 (33); 75 (43); 60 (34, 39, 50); 40 (40); 35–45 (49); 30–35 (58); 30 (51, 53); 25–20 (52); 20 (48); and 15 min (46). Two studies did not specify the duration—they mentioned, over 8–4 h per week (54, 59). One study did not report training duration (36).

All articles reported experiments lasting between 4 and 32 weeks. One study lasted 4 weeks (56); 5 lasted 6 weeks (37, 42, 45, 46, 50); 11 lasted 8 weeks (33, 35, 39–41, 43, 44, 48, 53, 58, 59); 2 lasted 9 weeks (51, 57); 1 lasted 10 weeks (34); 3 lasted 12 weeks (38, 49, 52); and 2 studies lasted 32 weeks (54, 55). One study did not report this aspect (36).

### 3.5. Outcome

The results were grouped based on the impact of core training on various skill-related physical fitness factors in professional soccer players.

#### 3.5.1. Effect of core training on power performance

In total, 17 of the 26 articles considered in this review showed results regarding the effect of core training on the overall power performance. Among these articles, 13 used the vertical jump test (33, 37, 38, 41, 44–46, 49, 51, 52, 55, 57, 59), nine used the standing long jump test (39, 41–43, 49, 51, 52, 54, 59), and one study used the throwing medicine ball test (52). The participants of these studies included university participants (33, 55), professional female participants (37), amateur participants (41–44, 46); semi-professional participants (59), junior participants (49), secondary participants (51), and elite participants (57); five studies did not report these levels (38, 39, 45, 52, 54). Most studies showed a significant increase in power performance after core training interventions; only four studies found little significant changes after the vertical jump test (37, 52, 57, 59, 60) and standing long jump test (59). Nine studies reported a comparison between experimental and control groups in the post-test (33, 37, 39, 43–45, 48, 52, 54). However, a few studies revealed significant differences between groups in vertical jump (35.93 ± 6.463 vs. 35.602 ± 5.572, *p* < 0.05) (45), (37.92 ± 15.02 vs. 31.02 ± 7.01, *p* < 0.05) (52); standing long jump (1.72 ± 0.12 vs. 1.43 ± 0.12, *p* < 0.05) (52), (2.69 ± 0.88 vs. 2.49 ± 0.89, *p* < 0.05) (54); and throwing medicine ball (4.52 ± 0.27 vs. 4.18 ± 0.42, *p* < 0.05) (52). In particular, one study involved three groups: dynamic core training (DCT), static core training (SCT), and routine training (TT). In the post-test, a significant difference could be observed only between DCT and TT (230.3 ± 8.6 vs. 220.3 ± 3.5, *p* < 0.05); SCT and TT (225 ± 5.3 vs. 220.3 ± 3.5, *p* < 0.05) (43).

#### 3.5.2. Effect of core training on speed performance

A total of 20 studies involved speed tests: three of them used a 20 m sprint test (42, 50, 57); one used a 20 m speed test (33); four used a 10 m sprint test (37, 42, 48, 50); one used a repeated sprint ability test (37); three used a 10 m dash test (38, 49, 59); two used a 40 m dash (38, 49); two used a 10 m speed test (39, 41); three used a 30 m speed test (39, 41, 51); one used a 35 m repeated sprint test (40); four used a 30 m sprint test (45, 46, 53, 54); one used a 30 m dash (59); one used a 100 m test (54); one used a 20 m starting run (55); and one study used a 40 m speed test (58). The participants in these studies included university students (33, 55), high school students (50), professional female participants (37), amateur participants (41, 42, 46, 52), semi-professional participants (59), junior participants (49), elite participants (48, 57), and secondary school participants (51); six studies did not report the level of participants (38–40, 45, 54, 58). Most studies showed a significant increase in speed performance after core training interventions. Only two studies found little significant changes in terms of the 10 m dash (59), 30 m dash (59), 30 m speed (39), and 10 m speed tests (39). Eight studies reported a comparison between the experimental and control groups in the post-test (33, 37, 39, 42, 45, 48, 52, 54). Only four studies reported significant difference between groups in the 20 m speed (2.80 ± 0.14 vs. 2.84 ± 0.19, *p* < 0.05) (33), 10 m sprint (2.07 ± 0.13 vs. 2.22 ± 0.13, *p* < 0.05) (42), 20 m sprint (3.41 ± 0.09 vs. 3.43 ± 0.24, *p* < 0.05) (52); 100 m speed (13.1 ± 1.3 vs. 13.75 ± 0.26, *p* < 0.05) (54), and 30 m speed tests (4.8 ± 1.1 vs. 5.2 ± 0.28, *p* < 0.05) (54).

#### 3.5.3. Effect of core training on balance performance

Six studies involved a balance test: one study used the Biodex balance system static balance test (34), two used a dynamic balance test (34, 35), one used the Y-balance test (35), one used a static balance test (56), one used the star excursion balance test (56), and two studies used the Flamingo balance test (45, 52); one study did not report the name of the balance test used (36). The study participants included male university participants (34, 35) and elite participants (36, 56); two studies did not report the level of participants (45, 52). The results of these studies showed that the balance test performance of the participants significantly improved after the core training intervention. Four studies reported a comparison between the experimental and control groups in the post-test (36, 45, 52, 61). Two studies reported significant differences between groups in the balance test (*p* < 0.05) (36) and Flamingo balance test (1.27 ± 0.25 vs. 2.92 ± 0.22, *p* < 0.05) (52).

#### 3.5.4. Effect of core training on agility performance

Sixteen studies used an agility test: five studies used the Illinois agility test (38, 46, 49, 53, 55), four used a *T*-test (38, 49, 57, 58), two used a zigzag test (42, 50), two used a 505 agility test (51, 59), two used an Arrowhead agility test (51, 59), one used a *V*-cut test (48), one used a *T*-Drill test (53), and one study used a Hexagon test (58). The study participants included university students (55), high school participants (50), amateur participants (42, 46, 53), semi-professional participants (59), junior participants (49), elite male participants (48, 57), and secondary school participants (51); two studies did not report the level of participants (38, 58). Most studies showed that core training can significantly improve agility performance. However, one study suggested little significant change in the Arrowhead and 505 agility tests after core training interventions. Additionally, three studies showed similar results: little significant increase was found in the *t*-test (57), hexagon test (58), and zigzag test (50). Only one study reported a comparison between experimental and control groups at post-test (48), and the results suggested little significant difference between groups in the *V*-cut [*d* = −0.56 95% CI (−1.89, 0.78), *p* = 0.370].

## 4. Discussion

This systematic review provides a comprehensive overview of the effects of core training on skill-related physical fitness among soccer players. Its main findings show that core training could improve power, speed, balance, and agility in soccer players. However, no evidence was provided regarding coordination and reaction time. Based on the above results, core training can be considered an effective intervention for soccer players. Following the framework of the “outcome” section, the theory behind the findings is explained in detail.

### 4.1. Effect of core training on power performance

The product of the body's strength and speed reflects power (62). Most coaches agree that many explosive tasks, including sprinting, jumping, throwing, and kicking, require power for successful performance (63, 64). This is because these sports demand high-speed movement and force generation (63, 65). Most of the above studies involving power tests have shown that core training has a positive effect on power performance. This could be explained by the core being intimately related to the lower limbs attached to the hip joint, and strong core muscles collecting more energy and reducing energy consumption, which plays a crucial role in stabilizing and transferring lower limb energy (61, 66). Meanwhile, owing to the strong core muscles, the participants demonstrated flexion of the hip, knee, and ankle when jumping, thus forming an overall stable body. Simultaneously, the power transmission of the upper limbs, trunk, and lower limbs was more coordinated (67). Sannicandro et al. (68) investigated 42 young basketball players and identified a significant difference in jump performance after 4 weeks of core training (2 times/week, 1 h/session). Bilici and Selçuk (69) identified that female volleyball players benefited from 8 weeks of core training (3 times/week, 1 h/session). Meanwhile, only one study involved an upper limb power test called the throwing medicine ball test while sitting, and also showed the positive impact of core training (70). The throw facilitates understanding how the force generated by the foot is transmitted to the hands. Evidently, there must be a substantial transfer of force through the core (29). The force generated by the larger movers of the lower extremities can be transferred to the arms because the stability generated by the anterior and posterior core muscles is provided by the lateral muscles opposing rotation. If the core is too weak to withstand the force, rotation is likely to occur, leading to a loss of energy and breakdown in the technique, which ultimately lowers the performance (29); a solid core transmits force with minimum rotation and energy loss (61). Additionally, several studies have demonstrated that strengthening the abdominal region increases the upper extremity pushing muscle force (70, 71). Parkhouse and Ball (72) supported the above theory that after 6 weeks of core exercise intervention (2 times/week, 45 min/session), a significant difference was observed in the TCM ball test.

### 4.2. Effect of core training on speed performance

Speed plays a critical role in soccer (73, 74); most studies in this review examined whether core training could improve the speed performance of soccer players. This may be explained by the theory that a stronger core enables the spine and pelvis to maintain stability (75). This improves the stability of the center of gravity during fast running and reduces fluctuations in it. With an increase in the stability and flexibility of the hip joint, the range of motion, stride, and stride frequency of athletes increases during the actual movement process (76). Bora and Daglioglu (77) investigated 18 young male volleyball players and identified that after 6 weeks of core training (3 times/week, 1 h/session), a significant difference was observed in the speed test. A similar positive effect was identified in another study after 4 weeks of core training (3 times/week) improved speed performance among 23 male students (78).

### 4.3. Effect of core training on balance performance

The significance of this balance is undervalued. Balance is a crucial factor in reducing the risk of injury (79). Nonetheless, soccer heavily relies on one-legged assistance in hazardous settings. Typically, players utilize one limb—the dominant one—to control the force and direction of the ball while dribbling, retaining ball possession, and kicking; the non-dominant limb provides the necessary stability to optimally execute the required technical maneuver (80). Therefore, high balance ability is essential for soccer players (81). The majority of the studies in this review evaluated whether core training can enhance balance. The abdominal and pelvic muscles constitute the segmental linkages between the upper and lower body and operate as the fulcrum in the core muscles, whereas the upper and lower body act as movable levers (82). Consequently, core stability can impact balance, physical activity, and performance. Instability or weakness in the core can also result in lower-body injuries and poor performance-related balance (83). Additionally, abrupt perturbations delivered to the body during a soccer game can potentially displace the body's center of gravity from its support foundation. To prevent loss of balance and falls, postural modifications are performed to reposition the center of gravity within the support base. These postural modifications necessitate core muscle activation to stabilize the lumbar spine (84–86). Therefore, Faries and Greenwood (83) concluded that “balance is derived from the core; a solid core equals good balance.” Sandrey and Mitzel (87) showed that 6 weeks of core training (3 times/week, 30 min/session) significantly improved balance performance among 13 track and field athletes. Dogan and Savaş (88) identified a significant difference in balance performance among basketball players after applying 8 weeks of core training (3 times/week, 45–60 min/session).

### 4.4. Effect of core strength training on agility performance

Agility refers to the capacity to rapidly alter body direction and position (89). Based on the information provided by the analyzed studies, it is evident that core training can improve soccer players' agility. A complicated process, including biomechanical, motor, sensory, and central nervous system components (90), may provide an explanation. The core could be considered the center of the kinetic chain in sports. Enhanced core muscles result in enhanced motor recruitment, neural recruitment, and neural adaptation (47). Thus, core strength, balance, and movement control maximize the function of the lower and upper extremities. Athletes' motor abilities, such as coordination, agility, speed, and balance in sports such as soccer should be predicted to improve as their core strength and stability improve (47). Meanwhile, rotations or direction changes are ubiquitous in agility assessments. Rotation indicates energy loss, which implies diminished performance. In core muscle groups, the external oblique, together with the hip and upper back muscles, creates and controls the rotation required to execute these actions (29). Therefore, the strong lateral core muscles not only facilitate a rotating action in a number of tasks but also resist rotational pressure in other activities (29). This can assist soccer players in improving their agility. Sighamoney et al. (91) showed that 4 weeks of core training (5 times/week) improved agility performance among badminton players. A similar result was found in other sports as well. Another study identified a significant difference in agility performance among runners after 4 weeks of core training (3 times/week) (92). Akçinar and Macit (93) suggested that 8 weeks of core training (3 times/week, 25–30 min) improved agility performance among male handball athletes.

## 5. Limitations

Despite this study providing evidence to assess the effect of core training on skill-related physical fitness among soccer players, the following limitations were noted. First, this review limited the search to databases containing predominantly English-language records; non-English studies were ignored. Second, existing studies have mainly compared routine training, lacking a comparison between core training and other innovative strength training regimen. Finally, current studies lack participant research on male professional soccer players.

## 6. Conclusion

This review shows that core training can improve the skill-related physical fitness of soccer players, including power, speed, balance, and agility; however, it could not accurately identify which physical components would benefit the most from core training. Moreover, the type of core training that can optimize skill-related physical fitness is still unclear. Additionally, research on other skill-related physical fitness in terms of coordination and reaction time is lacking in the extant literature. Therefore, researchers should investigate these gaps to improve the physical performance of soccer players.

## 7. Applications

The core is the anatomical and functional focal point and engine of the body. All movements emanate from the center of the body and are transmitted to the extremities. The core musculature differs from the limb musculature in that the core muscles frequently co-contract, hardening the torso to the point where all muscles become synergists. Theoretically, a strong core facilitates the passage of force from the lower to the upper body, with minimal energy loss in the torso. Therefore, this force can be delivered to the limbs more effectively, resulting in improved skill-related physical fitness performance.

Based on these 26 studies, this review suggests that core training should be incorporated into the daily training of soccer players, with a minimum frequency and length of 15 min per training, twice per week, for 4 weeks.

## Data availability statement

The original contributions presented in the study are included in the article/supplementary material, further inquiries can be directed to the corresponding author.

## Author contributions

SL drafted the article. SL and KS provided data interpretation. LZ critically revised the article and gave the final approval. All authors read and approved the final manuscript.
